# DXM, CYP2D6-inhibiting antidepressants, piracetam, and glutamine: proposing a ketamine-class antidepressant regimen with existing drugs

**DOI:** 10.3389/fpsyt.2026.1751605

**Published:** 2026-03-17

**Authors:** Ngo Cheung

**Affiliations:** Cheung Ngo Medical Limited, Hong Kong, Hong Kong SAR, China

**Keywords:** CYP2D6-inhibiting antidepressants, depression, DXM, glutamatergic, glutamine, ketamine, piracetam

## Abstract

Rapid-acting antidepressants show that mood can lift within hours when glutamatergic circuits shift from an “NMDA-dominant” to an “AMPA-dominant” state. Intravenous ketamine achieves this flip but is hampered by dissociation and logistics, while dextromethorphan + bupropion (Auvelity^®^) primarily supplies the initial NMDA blockade and yields slower, less durable benefit. We hypothesize that a fully oral, low-cost, four-component regimen may be able to approximate ketamine’s full plasticity cascade (1) dextromethorphan (DXM) for NMDA antagonism; (2) a potent CYP2D6 inhibitor (fluoxetine, paroxetine, or high-dose duloxetine) to prolong DXM exposure; (3) piracetam as an AMPA positive allosteric modulator; and (4) micronized L-glutamine to restore presynaptic glutamate pools and buffer against excitotoxicity. Preclinical evidence supports mechanistic synergy along the same axis, but the full combination remains untested in humans. This hypothesis warrants formal preclinical and clinical evaluation.

## Introduction

The past decade has revealed that the fastest antidepressant relief comes not from tweaking serotonin but from jump-starting glutamatergic plasticity. Ketamine’s single-dose success proves the concept, yet its dissociation, abuse potential, and infusion logistics keep it out of reach for most patients. Meanwhile, the oral agent Auvelity (dextromethorphan + bupropion) shows that we can deliver an NMDA-blocking “spark” in pill form, but its benefits arrive more slowly and fade more quickly than ketamine’s—likely because the combination never fully flips the synapse into the AMPA-dominated state that drives lasting circuit repair. Here we propose a four-component, all-oral regimen designed to recreate ketamine’s full plasticity cascade without IV lines or street-drug baggage: (1) dextromethorphan for NMDA antagonism, (2) a potent CYP2D6 inhibitor such as fluoxetine to keep dextromethorphan active, (3) the inexpensive AMPA positive allosteric modulator piracetam to amplify downstream signaling, and (4) dietary L-glutamine to replenish presynaptic glutamate stores and buffer excitotoxic risk. Each element is already marketed, inexpensive, and well characterized, yet together they form a mechanistically coherent strategy to deliver rapid, sustained antidepressant action. Here we hypothesize and outline a four-component, all-oral regimen intended to recreate ketamine’s plasticity cascade to the extent possible with marketed agents.

## How ketamine ignites AMPA-fueled neuroplasticity to lift depression

**Figure 1:**
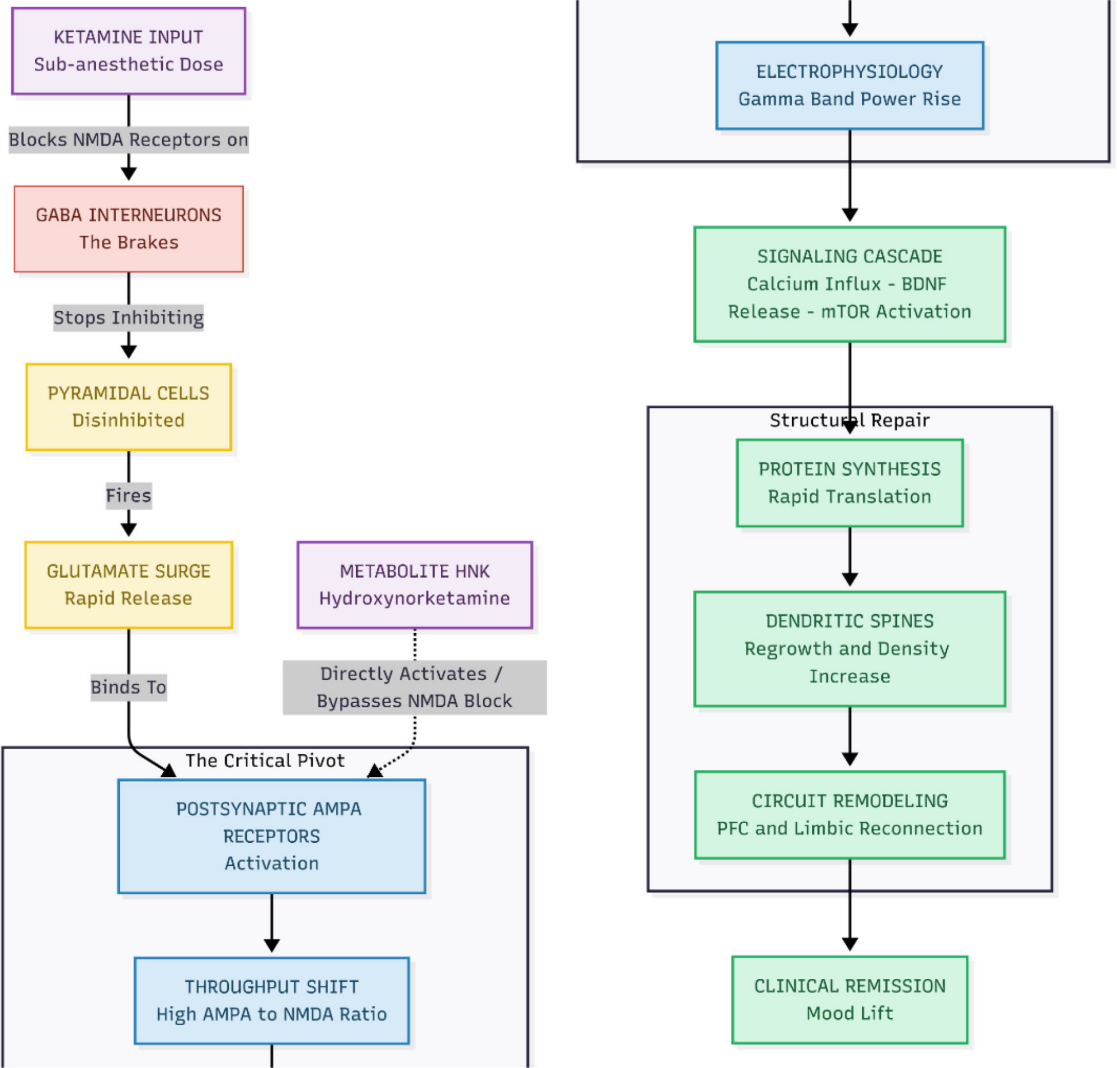
Ketamine initiates its antidepressant effect through the disinhibition trigger, by blocking NMDA receptors specifically on GABA interneurons—the brain’s natural “brakes.” This disinhibition unleashes a burst of activity in excitatory pyramidal neurons, flooding synapses with glutamate and creating the initial spark. As NMDA receptors are blocked or subdued, the excess glutamate is funneled through AMPA receptors, marking The AMPA Dominance phase, where synaptic signaling shifts toward a “High AMPA/Low NMDA” throughput. Studies confirm that blocking AMPA receptors nullifies Ketamine’s antidepressant action, highlighting their central role. Finally, Structural Restoration occurs as AMPA activation leads to calcium influx, which triggers BDNF release and mTOR activation. This cascade rapidly synthesizes new proteins, regrowing dendritic spines lost to stress—and the resulting physical repair of neural circuits closely tracks with the observed clinical improvement in mood.

Ketamine’s antidepressant story begins at the NMDA receptor (**Figure 1**). At sub-anesthetic doses the drug preferentially blocks NMDA receptors that sit on inhibitory GABA interneurons in the prefrontal cortex and hippocampus. Momentarily silencing these “brakes” disinhibits nearby pyramidal cells and triggers a burst of glutamate release (1). This glutamate surge is not simply biochemical noise; in patients it coincides with mood lift that can appear within hours and last for days—an effect unmatched by conventional monoaminergic drugs that require weeks (2). Yet the NMDA story is only half the plot: other NMDA antagonists (e.g., memantine or MK-801) fail to reproduce ketamine’s robust clinical benefit, and the less potent NMDA-blocking R-enantiomer of ketamine actually shows the stronger and longer-lasting antidepressant activity (3). Those clues point beyond NMDA blockade alone.

The glutamate pulse set loose by ketamine lands squarely on postsynaptic AMPA receptors. Rodent work shows that blocking AMPA receptors with NBQX abolishes both the immediate and the sustained behavioral antidepressant effects of ketamine, while positive AMPA modulators (“AMPAkines”) mimic or amplify the response (4, 5). Electrophysiology and EEG demonstrate a rapid rise in AMPA-mediated currents and γ-band power, and within an hour the surface expression of GluA1/GluA2 subunits is up-regulated in prefrontal and hippocampal synapses (6). In short, ketamine appears to flip the glutamatergic balance from “NMDA dominant” to “AMPA dominant, “ a shift sometimes summarized as increasing the AMPA/NMDA throughput ratio, which is thought to be the proximal signal for its antidepressant action. Not every element of ketamine’s pharmacology is necessarily required for antidepressant action; NMDA blockade on GABA interneurons appears necessary to trigger the glutamate burst, whereas full AMPA engagement and downstream BDNF/mTOR signaling appear critical for sustained effects, while dissociation itself is likely incidental.

**Figure 2:**
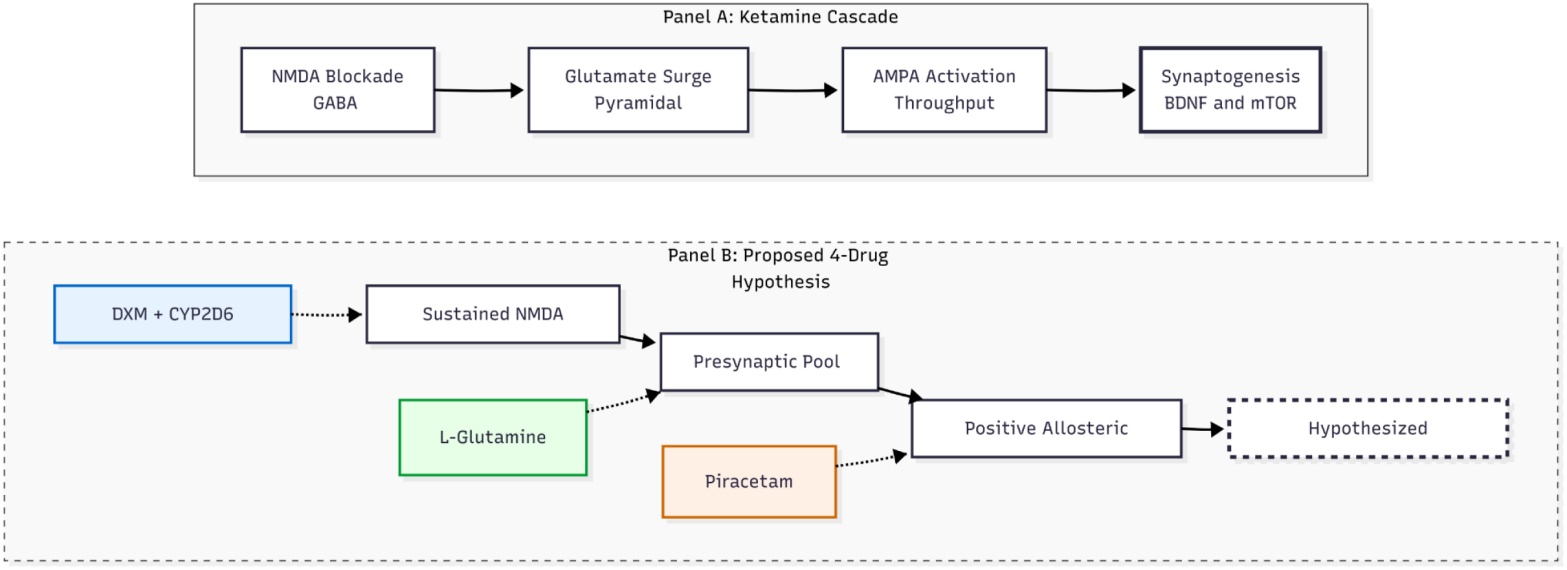
Mechanistic decomposition schematic. Panel **(A)** illustrates the established biological cascade of intravenous ketamine: NMDA blockade on GABAergic interneurons disinhibits pyramidal neurons, causing a glutamate surge that activates AMPA receptors, triggering downstream BDNF/mTOR signaling and synaptogenesis. Panel **(B)** maps the hypothesized four-drug regimen to these distinct mechanistic steps. Dextromethorphan (DXM), potentiated by a CYP2D6 inhibitor, provides sustained NMDA blockade; L-Glutamine supports the glutamate pool to prevent depletion; and Piracetam acts as a Positive Allosteric Modulator (PAM) to facilitate AMPA throughput, theoretically approximating the final neuroplasticity endpoint.

When AMPA receptors fire, the resulting calcium rush prompts the release of brain-derived neurotrophic factor (BDNF) and switches on the mTOR pathway. That, in turn, jump-starts rapid protein synthesis, dendritic spine growth, and circuit remodeling that repairs stress-related synaptic loss (1, 6). Within about 24 h, spine density and the functional connections linking prefrontal and limbic regions rebound, and the extent of this rebound tracks with how long symptom relief lasts. Remarkably, the ketamine metabolite hydroxynorketamine (HNK) can reproduce the same AMPA-, BDNF-, and mTOR-dependent effects without blocking NMDA receptors or causing dissociation, pointing to the possibility of safer next-generation treatments (3). The evidence indicates that ketamine alleviates depression by temporarily inhibiting the NMDA receptor and activating the AMPA receptor, thereby reactivating the brain’s inherent capacity for adaptive neuroplasticity (**Figure 2**).

## Auvelity: delivering the NMDA spark but missing the full AMPA flame

**Figure 3:**
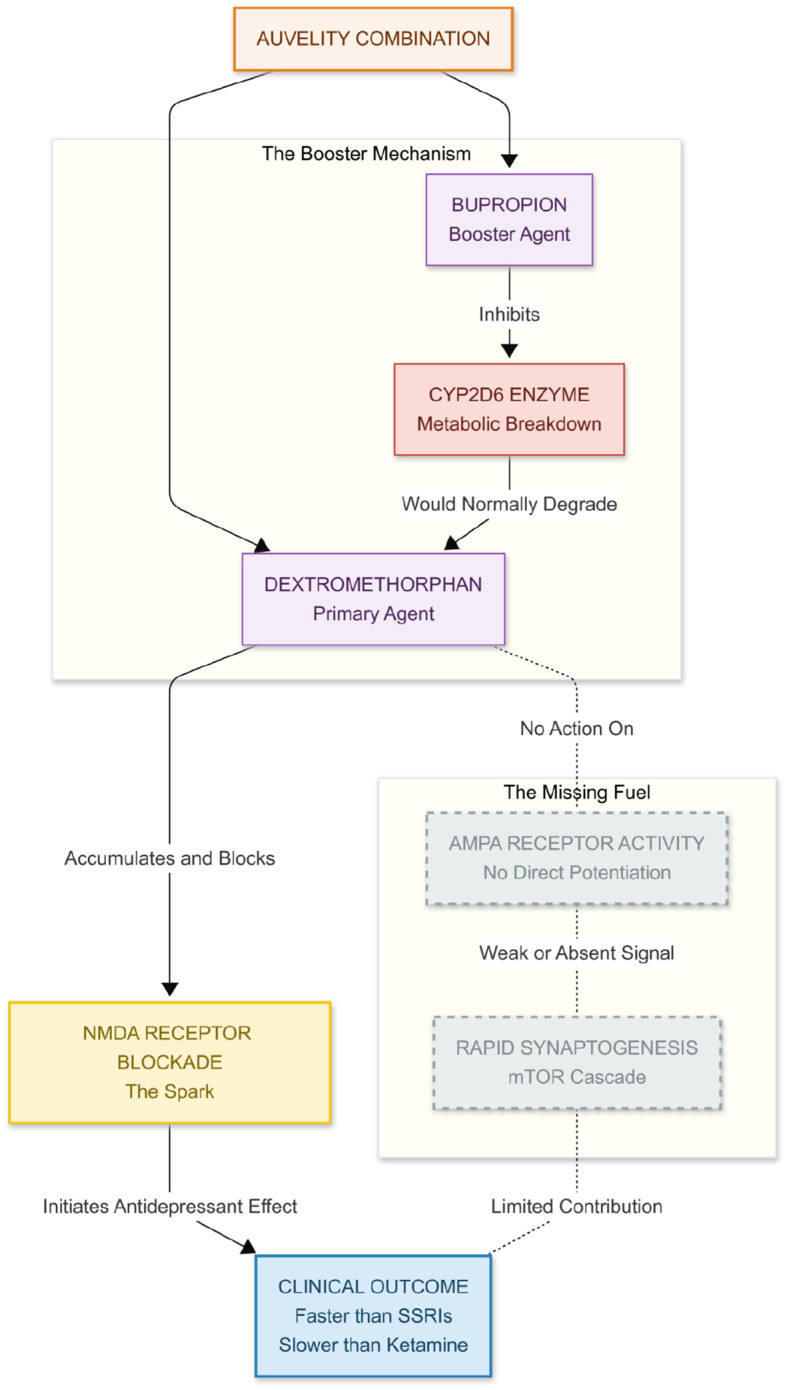
Auvelity achieves its effect through the kinetic boost, using a pharmacokinetic strategy where Bupropion not only contributes to mood regulation but also inhibits the CYP2D6 enzyme, preventing the rapid breakdown of Dextromethorphan and allowing it to reach therapeutic concentrations in the brain—levels that would otherwise be unachievable. This sets the stage for The NMDA Spark, as Dextromethorphan, like Ketamine, acts as an NMDA antagonist, reducing NMDA signaling and providing the initial antidepressant “spark, “ which likely accounts for Auvelity’s faster onset compared to traditional antidepressants. However, The Missing AMPA Flame highlights a key limitation: unlike Ketamine, Dextromethorphan does not trigger a glutamate surge or directly engage AMPA receptors. Without this critical “fuel, “ the cascade leading to rapid synaptogenesis and structural neural repair may be weaker, potentially explaining Auvelity’s comparatively reduced efficacy and speed.

Auvelity^®^—the fixed-dose combination of dextromethorphan and bupropion—appears to replicate solely the initial phase of ketamine’s antidepressant cascade (**Figure 3**). Dextromethorphan, an uncompetitive NMDA-receptor antagonist like ketamine, reduces NMDA-mediated signalling, while bupropion slows its breakdown by inhibiting CYP2D6, thereby extending exposure (7). Whether the combination also produces the secondary rise in AMPA-receptor activity believed to drive ketamine-related synaptogenesis and prolonged mood benefits remains an open question. Dextromethorphan has no intrinsic AMPA-potentiating action, and neither ingredient directly augments AMPA currents. In other words, Auvelity may supply the NMDA-blocking “spark” without the AMPA “fuel.” This pharmacological gap could help explain why Auvelity acts faster than classic monoaminergic antidepressants yet still lags behind ketamine in both onset and effect size, and why the durability of its benefit is still being studied (7).

## Using CYP2D6 inhibitors to keep dextromethorphan active without bupropion

When the bupropion half of Auvelity is unavailable, the simplest way to keep dextromethorphan (DXM) from being chewed up too quickly is to pair it with another psychotropic that blocks the same metabolic “off-ramp, “ CYP2D6. Fluoxetine and paroxetine stand out because they are high-affinity, mechanism-based inhibitors that can push extensive metabolizers into the poor-metabolizer range within days (8, 9). In practice, adding 20 mg of fluoxetine or 10–20 mg of paroxetine once daily would be expected to raise DXM exposure to a degree comparable with—or greater than—the 105 mg of bupropion found in Auvelity, while simultaneously providing their own SSRI antidepressant activity. Duloxetine offers a middle-ground: it is only a moderate inhibitor, yet at 60 mg daily can still curb CYP2D6 enough to meaningfully boost DXM while delivering dual serotonin–norepinephrine re-uptake benefits (10). Sertraline could work in a pinch, but only at ≥100 mg, a dose that many patients find activating (11).

Antipsychotics present a more unconventional—but pharmacologically sound—backup plan. Low doses of perphenazine or thioridazine inhibit CYP2D6 with Ki values in the same ballpark as the laboratory reference inhibitor quinidine, suggesting that even 2–4 mg of perphenazine at bedtime could “protect” DXM throughout the day (12). Risperidone is gentler, acting as a mild inhibitor at routine doses, yet it might suffice in patients who already take a mood-stabilizing atypical and simply need a modest DXM boost (13). Whichever substitute is chosen, clinicians must remember that blocking CYP2D6 is a double-edged sword: it enhances DXM’s rapid-acting antidepressant punch but can also elevate plasma levels of any co-prescribed β-blockers, opioids, or tamoxifen (14). Close monitoring—especially during the first two weeks—keeps the strategy safe, letting DXM do its NMDA-modulating job while its “pharmacokinetic bodyguard” stands watch.

CYP2D6 genotype markedly affects DXM exposure. Poor metabolizers (~5–10% of Caucasians, lower in Asian populations) achieve high DXM levels naturally, while ultra-rapid metabolizers may require stronger inhibition or higher doses. Fluoxetine and paroxetine produce time-dependent inhibition that builds over days. Therefore, genotyping (where available), conservative starting doses, and close clinical monitoring during the first 1–2 weeks are essential.

## How budget- friendly piracetam can supercharge auvelity’s antidepressant spark

Auvelity (dextromethorphan + bupropion) relieves depression by two complementary moves: bupropion boosts cortical catecholamine and glutamate release, while dextromethorphan lowers background “static” at NMDA receptors so that meaningful glutamatergic bursts come through more clearly (15). What the combo does ​not​ directly address is the final hand-off of that glutamate signal to AMPA receptors, the fast synaptic switches that trigger BDNF release and downstream mood-repair cascades. Piracetam—and several other inexpensive AMPA-positive allosteric modulators (e.g., aniracetam, CX-series ampakines)—can fill this gap. In aged animals piracetam increases AMPA receptor density and enhances AMPA-mediated currents, thereby strengthening long-term potentiation and synaptic plasticity (16, 17). Layering a cheap, well-tolerated dose of piracetam (1–3 g/day oral; 18) onto standard Auvelity could therefore push more of the liberated glutamate through a “wide-open” AMPA channel, theoretically translating into a quicker and more robust lift in mood, energy, and cognitive drive.

**Figure 4:**
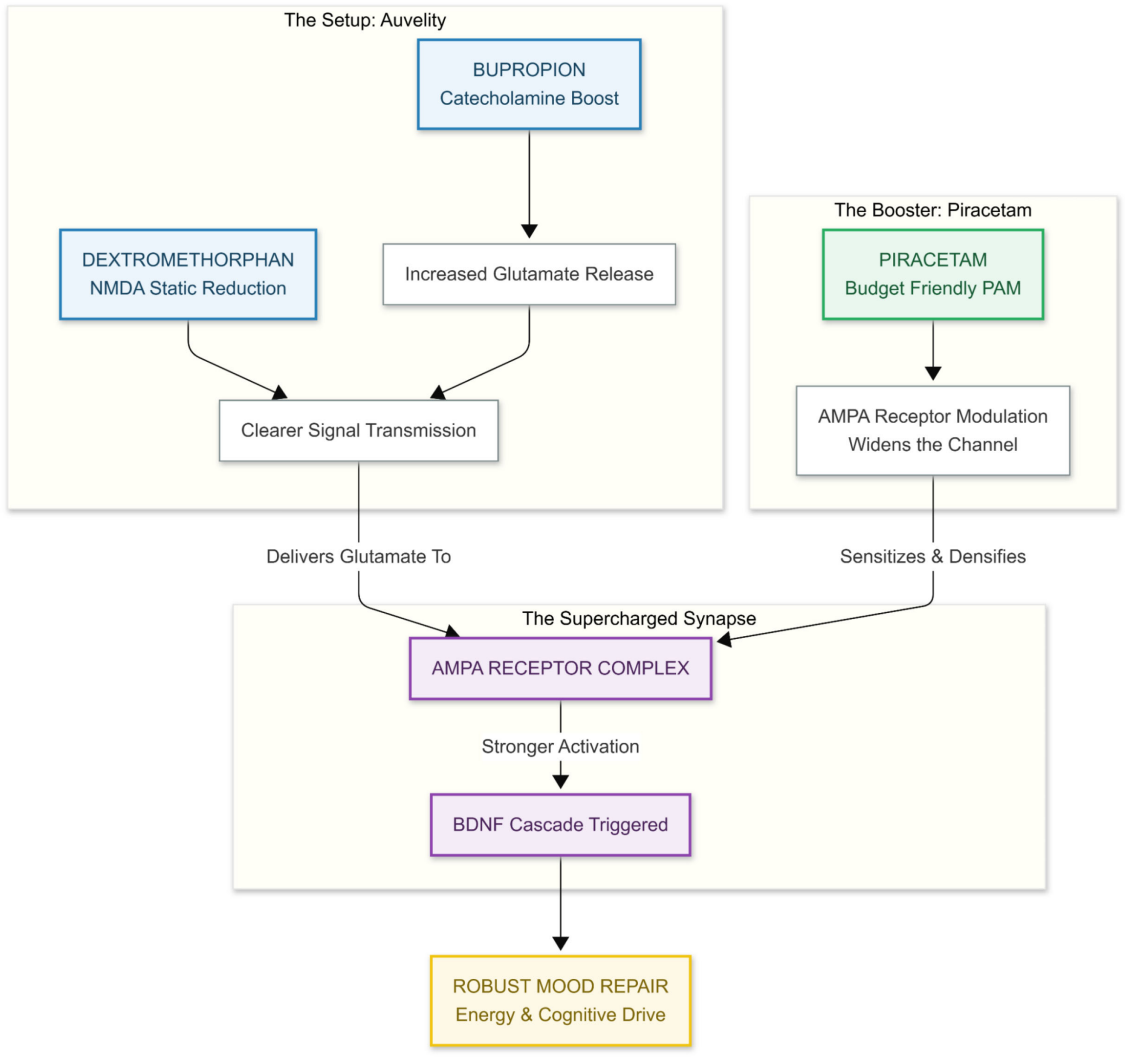
The auvelity “spark” primes the brain for transformation by combining bupropion, which boosts glutamate release, with dextromethorphan, which clears NMDA receptor “static,“ producing a clean, high-fidelity glutamate signal ready for transmission. However, this signal requires a sensitive target, which is where The Piracetam “Fuel” comes in—acting as an AMPA Positive Allosteric Modulator (PAM), Piracetam increases AMPA receptor density and keeps the channels “wide open, “ ensuring the glutamate signal is fully captured rather than dissipated. The synergy between the clean signal provided by Auvelity and the sensitized receptors enabled by Piracetam leads to The Supercharged Result: a potent release of BDNF that enhances synaptic plasticity and accelerates mood repair, theoretically delivering a faster lift in energy, cognition, and emotional resilience than Auvelity alone.

From a practical standpoint the strategy is attractive (**Figure 4**): piracetam is generic in most countries, costs only a few dollars per week, and carries a side-effect profile that is gentler than many vitamins (18). Because it lacks significant hepatic metabolism or protein binding, drug–drug interactions with Auvelity are unlikely, and the main safety considerations are mild insomnia or jitteriness—signs that the AMPA gain may simply need dialing back. Until head-to-head trials are run, clinicians could consider a cautious “AMPA-priming” add-on in patients who have a partial response to Auvelity alone, monitoring sleep and anxiety just as they would when titrating bupropion. In short, Auvelity may provide the NMDA “spark, “ but a low-cost AMPA PAM like piracetam can help the antidepressant flame catch—and stay lit.

Piracetam got the nod here because it’s the easiest to get and the most thoroughly studied of the racetam family. Sure, aniracetam and some of the CX-series compounds hit AMPA harder in cell assays and certain animal work, but piracetam brings something none of them can match right now: decades of real-world human safety data, rock-bottom pricing, almost no hepatic metabolism to worry about, and you can source it practically anywhere. The honest trade-off is that its AMPA potentiation in people is on the modest side compared to the newer experimental ampakines — that’s a deliberate concession to practicality, not a claim that it’s the strongest tool for the job. If a future revision of this stack calls for more firepower at the receptor and the side-effect profile holds up, swapping in a more potent agent would be straightforward.

## How glutamine supercharges the new glutamatergic stack

**Figure 5:**
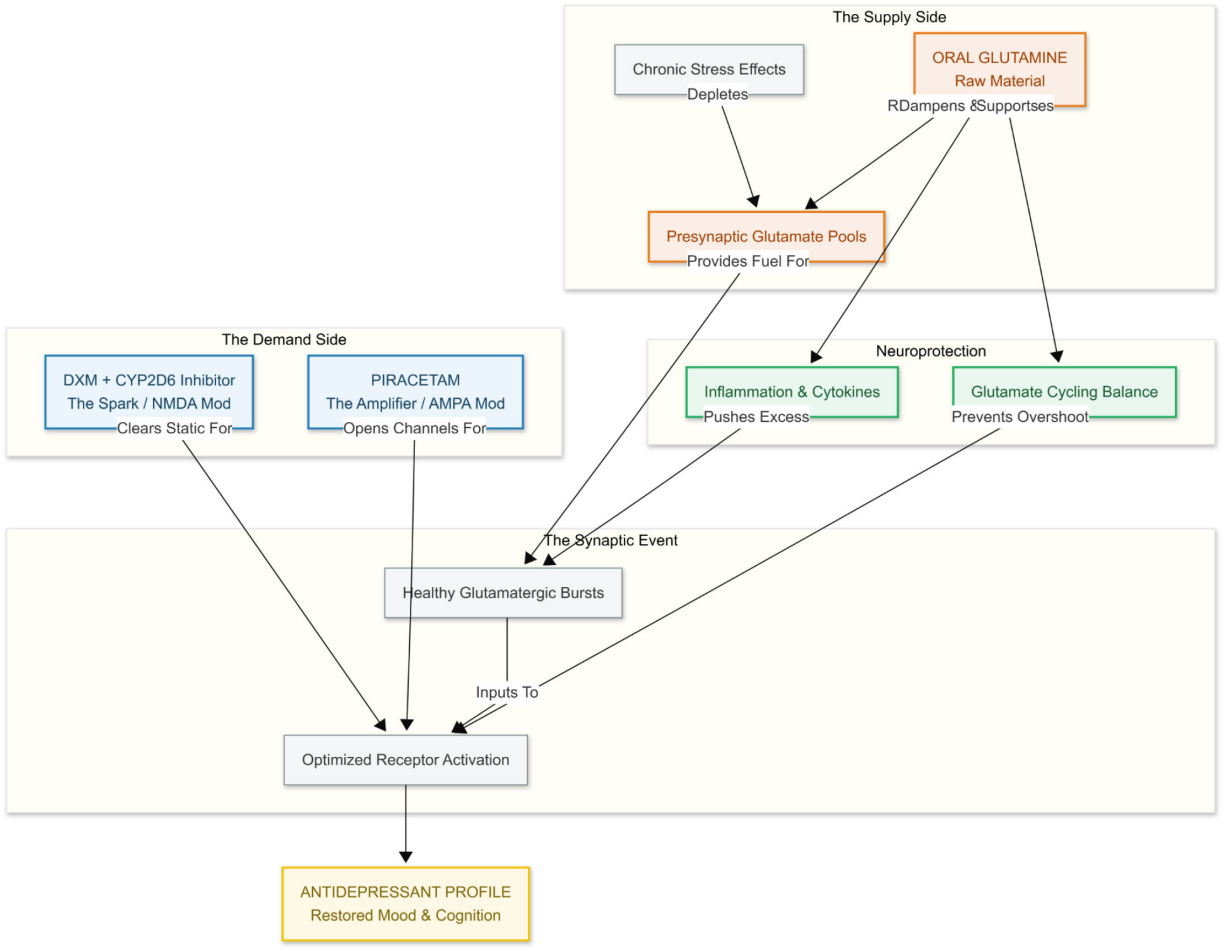
Refilling the tank is essential because chronic stress depletes the brain’s presynaptic glutamate reserves, and without sufficient glutamine, there’s simply no fuel to generate healthy glutamatergic bursts. Oral Glutamine replenishes this supply, providing the raw material needed to sustain neurotransmission and potentially replacing the need for Bupropion’s glutamate-releasing effect. As Fueling the Machinery, Glutamine becomes critical in a stack that includes DXM, which clears inhibitory blocks, and Piracetam, which enhances AMPA receptor throughput—both of which increase demand without supplying the signal itself. Glutamine ensures there’s actual neurotransmitter available to be transmitted through the enhanced circuit. Finally, as The Safety Valve, Glutamine adds a protective layer by buffering against excitotoxicity—a known risk when combining NMDA modulation with AMPA amplification. It helps regulate inflammation-driven glutamate spikes and stabilizes cycle dynamics, preventing overshoot and neuronal damage, thereby keeping the stack both effective and sustainable.

Adding oral glutamine to the new regime (i.e., dextromethorphan prolonged by a CYP2D6 inhibitor plus the AMPA-priming piracetam) can replenish the raw material the brain needs to fire healthy glutamatergic bursts. Chronic stress depletes presynaptic pools of glutamate, blunting excitatory postsynaptic currents in the medial prefrontal cortex and flattening mood; in rodents, just one to two weeks of dietary glutamine reverses these deficits, normalizes glutamate and glutamine levels, and produces a clear-cut antidepressant-like behavioral profile (19, 20). By topping up intracellular glutamine, we ensure that dextromethorphan’s NMDA-modulating “spark” and piracetam’s AMPA-opening “amplifier” actually have enough neurotransmitter fuel to work with—something especially important now that bupropion (an inhibitor of stimulus-evoked glutamate release) is no longer in the mix (21). In short, glutamine can restore the supply side of the synapse while the rest of the stack optimizes demand (**Figure 5**).

Glutamine also seems to protect the brain. When inflammation is brewing, microglial signals like TNF-α and IL-1β push neurons to crank up glutaminase, flooding the space between cells with too much glutamate (22). Large supplemental doses of glutamine actually dial that problem back: they lower spinal-fluid glutamate in people with mitochondrial encephalopathy (23) and blunt the cognitive hit of cytokines in Alzheimer’s mouse models (24). Keeping glutamate cycling smoothly this way could cut down on the excitotoxic “overshoot” that can happen when NMDA blockers and AMPA boosters are used together.

Glutamine’s dual role as precursor and physiological buffer is particularly important in a multi-component glutamatergic stack, helping to prevent the very glutamate-surge overshoot that could otherwise occur when NMDA blockade and AMPA amplification are combined.

## Safety considerations and side effects

The four-component regimen described here—recently designated the Cheung Glutamatergic Regimen (CGR) in clinical reports—invites reasonable scrutiny around polypharmacy burden, pharmacokinetic unpredictability, and the possibility that stacking multiple glutamatergic mechanisms could push cumulative excitatory drive beyond a safe threshold. The most salient pharmacological risk is serotonin toxicity. Dextromethorphan carries weak serotonin-reuptake transporter inhibitory activity on its own, and when a potent CYP2D6 inhibitor such as fluoxetine or paroxetine is added, plasma concentrations of both dextromethorphan and its active metabolite dextrorphan can rise substantially. That said, naturalistic clinical use of the CGR, or close variants of it, has now been documented across more than fifty patients presenting with a range of refractory psychiatric conditions—treatment-resistant depression, bipolar depression, treatment-resistant obsessive-compulsive disorder, trauma-related syndromes, and somatic symptom disorders—and the overall tolerability profile has been favourable when the regimen is introduced with conservative titration and patients are monitored closely (25, 26).

Side effects reported to date have been predominantly mild and self-limiting, most appearing within the first one to two weeks of treatment. Roughly fifteen percent of patients experienced jitteriness or a fine tremor, and about ten percent showed mild autonomic arousal or transient tachycardia. Sleep disturbances—insomnia or somnolence—varied with the timing of dosing. Gastrointestinal discomfort was occasionally noted at higher glutamine doses, and brief hypomanic activation surfaced in a small number of patients on the bipolar spectrum. A handful of individuals developed mild, pre-syndromal serotonin-toxicity features such as trembling, sweating, or agitation, but in every instance these signs resolved quickly—typically within forty-eight hours—once the CYP2D6 inhibitor was reduced or temporarily paused. No case progressed to a full serotonin syndrome or required hospitalisation. Clinically meaningful dissociation was not observed at therapeutic dextromethorphan doses ranging from 15 to 120 mg per day. It is also worth noting that L-glutamine may serve as a physiological buffer against glutamate overshoot or excitotoxicity, given its role as a regulated precursor in the glutamate–glutamine cycle (26).

## Mitigation strategies

A set of mitigation strategies (**Table 1**) has been refined through ongoing real-world use of the regimen (25, 26). First, treatment should begin at low doses and be titrated slowly, guided by symptoms: an initial bedtime dose of dextromethorphan at 15 to 30 mg paired with a low-dose CYP2D6 inhibitor—for instance, fluoxetine 10 mg, paroxetine 10 mg, or duloxetine 30 to 60 mg—avoids early exposure to full-strength enzymatic blockade. Second, the components should be added sequentially rather than all at once. The two-drug core of dextromethorphan plus a CYP2D6 inhibitor is started first; after one to two weeks, once tolerability is established, piracetam is introduced at a starting dose of 600 mg per day, followed by micronized L-glutamine at 500 mg to 2 g per day only when there is a specific clinical rationale such as fatigue or stress-related glutamate depletion. Third, the choice of CYP2D6 inhibitor should follow a hierarchy that reflects patient vulnerability. Moderate or reversible agents—duloxetine, bupropion, or low-dose tricyclics—are preferable in sensitive individuals, including those on the bipolar spectrum or those already taking several medications, because their effects can be reversed more readily. Potent, longer-acting inhibitors like paroxetine or high-dose fluoxetine should be reserved for patients who have already demonstrated robust tolerability and should be used under close supervision.

**Table 1:** Mitigation strategies and clinical recommendations for safe implementation of the proposed regimen.

Strategy	Recommendation	Rationale/additional notes
Initiation & Titration	Start low, go slow, symptom-led: DXM 15–30 mg at bedtime + low-dose CYP2D6 inhibitor (fluoxetine 10 mg, paroxetine 10 mg, or duloxetine 30–60 mg). Avoid full-strength potent inhibitors initially.	Minimises early overstimulation
Sequential Addition	Begin with 2-drug core (DXM + CYP2D6 inhibitor) for 1–2 weeks, then add piracetam (start 600 mg/day), followed by micronized L-glutamine (500 mg–2 g/day) only if clinically indicated (e.g., fatigue, stress-related depletion).	Allows tolerability assessment before adding components
Inhibitor Selection Hierarchy	Prefer moderate/reversible agents (duloxetine, bupropion, low-dose tricyclics) in sensitive patients (bipolar spectrum, polypharmacy). Reserve potent irreversible inhibitors (paroxetine, high-dose fluoxetine) for robust responders under close supervision.	Easier reversibility and lower risk in vulnerable populations
Dosing Optimisation	Default to nighttime DXM to minimise daytime effects. Use split dosing (morning + bedtime) for afternoon rebound without increasing total daily dose.	Improves tolerability and adherence
Monitoring	Weekly (or more frequent initially) clinical assessment with MADRS (or Y-BOCS for OCD), vital signs, side-effect checklist, and active screening for early overstimulation/serotonin toxicity (agitation, tremor, clonus, hyperreflexia, hyperthermia) or mood activation.	Early detection and rapid intervention
Response to Overstimulation	Pause the CYP2D6 inhibitor for 2–3 days until symptoms resolve, then restart at approximately half dose. CYP2D6 genotyping strongly recommended when available.	Rapid, reversible management
Contraindications & Cautions	Avoid in untreated bipolar I disorder (without adequate mood stabilisation), active seizure disorders, or concomitant MAOIs/strong serotonergics. Use caution in adolescents, elderly patients, and those on multiple CYP2D6 substrates.	Prevents high-risk combinations

Fourth, dextromethorphan is best dosed at night by default to reduce daytime side effects, though split dosing across morning and bedtime can address afternoon symptom rebound without raising the total daily amount. Fifth, monitoring should be intensive at the outset—weekly or more frequent clinical assessments using standardised instruments such as the Montgomery–Åsberg Depression Rating Scale or the Yale–Brown Obsessive Compulsive Scale for OCD, along with vital signs, a structured side-effect checklist, and active screening for early signs of overstimulation or serotonin toxicity, including agitation, tremor, clonus, hyperreflexia, and hyperthermia.

In cases where mood activation or serotonergic overstimulation does emerge, the recommended response is to pause the CYP2D6 inhibitor for two to three days until symptoms settle, then reinstate it at roughly half the previous dose. CYP2D6 genotyping is strongly encouraged whenever it is available, as it allows dosing to be individualised based on metaboliser status rather than relying solely on clinical observation. Finally, certain populations and medication combinations warrant particular caution or represent outright contraindications. The regimen should be avoided in patients with untreated bipolar I disorder who lack adequate mood stabilisation, in those with active seizure disorders, and in anyone concurrently taking monoamine oxidase inhibitors or other strongly serotonergic agents. Extra care is also warranted in adolescents, elderly patients, and individuals already receiving multiple drugs metabolised through CYP2D6.

## Preclinical and clinical feasibility

Regarding preclinical and clinical feasibility, a point raised during peer review, it bears emphasis that the full CGR and all possible two- and three-drug subsets within it lend themselves readily to evaluation in standard rodent paradigms used to model depression and OCD-like behaviour. Chronic mild stress, the forced-swim test, sucrose preference, and marble-burying assays could all be employed, with behavioural outcomes supplemented by electrophysiological measures such as long-term potentiation and gamma-band power, as well as histological endpoints including dendritic spine density and brain-derived neurotrophic factor expression. Such preclinical work could and should precede larger human studies. Crucially, however, the path to controlled trials is not starting from zero: a meaningful body of naturalistic human experience already exists from outpatient settings, providing preliminary signals of both efficacy and safety that justify progression to more rigorous designs (25, 26).

## Limitations

Despite the encouraging early signals—rapid symptom improvement and generally acceptable tolerability—that have come out of uncontrolled naturalistic case series and open clinical reports (25, 26), the evidence base remains preliminary in important respects. No randomised, double-blind, placebo-controlled trial of the full four-drug combination has been carried out to date. The pharmacokinetic landscape is inherently unpredictable, particularly given the wide population variability in CYP2D6 activity, and the potential for additive central nervous system stimulation across the regimen’s components cannot be dismissed on the basis of open-label experience alone. Drug–drug interactions pose a genuine concern in patients who are already on multiple medications, a scenario that is common in the treatment-resistant populations most likely to be offered this regimen. Long-term safety data beyond approximately nine months are not yet available. For all of these reasons, larger, well-controlled, multicentre trials are essential before firm conclusions about the regimen’s risk–benefit profile can be drawn. Ideally, such trials would stratify participants by CYP2D6 genotype to disentangle pharmacokinetic sources of variability from true differences in pharmacodynamic response.

## Falsifiable predictions

A strength of the present hypothesis is that it generates specific, testable predictions whose failure would either refute or force substantial revision of the underlying model. If the proposed mechanism is correct, the regimen should produce statistically significant improvement on the Montgomery–Åsberg Depression Rating Scale—or on the Yale–Brown Obsessive Compulsive Scale in the case of OCD—within twenty-four to seventy-two hours, and certainly within two weeks, at a magnitude that exceeds what is achievable with dextromethorphan–bupropion (Auvelity) alone. Electroencephalographic or magnetoencephalographic recordings should reveal increased gamma-band power and a shift in the AMPA-to-NMDA throughput ratio comparable to what is observed following subanesthetic ketamine infusion. Peripheral brain-derived neurotrophic factor levels should rise, or neuroimaging should demonstrate evidence of rapid synaptogenesis, after one to two weeks of treatment. In animal models, antidepressant-like and anti-compulsive-like behavioural effects produced by the regimen should be abolished when an AMPA receptor antagonist such as NBQX is co-administered, confirming that the therapeutic signal depends on intact AMPA-mediated signalling. Negative results on any of these endpoints would meaningfully weaken the rationale for the regimen as it is currently conceived and would necessitate a fundamental rethinking of the model.

## Summary

The central hypothesis advanced here is that the Cheung Glutamatergic Regimen—which pairs pharmacokinetic prolongation of dextromethorphan through CYP2D6 inhibition with AMPA receptor facilitation via piracetam and glutamate precursor repletion via L-glutamine—may approximate the full rapid-acting plasticity cascade triggered by ketamine more closely than dextromethorphan–bupropion alone, while retaining the practical advantages of an entirely oral, low-cost, and widely accessible treatment. Preliminary naturalistic clinical experience supports both the feasibility and the promising efficacy of this approach across several refractory psychiatric conditions, with a safety profile that appears manageable when the optimised titration and monitoring framework described above is followed (25, 26). Should these early observations be confirmed through a rigorous stepwise research programme—moving from preclinical models through controlled dose-optimisation studies to randomised phase-two trials comparing the regimen head-to-head against Auvelity, with primary endpoints of rapid symptom change on validated rating scales and durability of response at four to twelve weeks—this strategy could meaningfully broaden access to ketamine-class therapeutic benefit for patients who currently have no realistic path to it outside of specialised infusion clinics.

## Data Availability

The original contributions presented in the study are included in the article/supplementary material. Further inquiries can be directed to the corresponding author.

## References

[1] Duman RS Aghajanian GK . Synaptic dysfunction in depression: Potential therapeutic targets. Science. (2012) 338:68–72. doi: 10.1126/science.1222939, PMID: 23042884 PMC4424898

[2] Berman RM Cappiello A Anand A Oren DA Heninger GR Charney DS . Antidepressant effects of ketamine in depressed patients. Biol Psychiatry. (2000) 47:351–4. doi: 10.1016/S0006-3223(99)00230-9, PMID: 10686270

[3] Zanos P Moaddel R Morris PJ Georgiou P Fischell J Elmer GI . NMDAR inhibition-independent antidepressant actions of ketamine metabolites. Nature. (2016) 533:481–6. doi: 10.1038/nature17998, PMID: 27144355 PMC4922311

[4] Maeng S Zarate Jr CA Du J Schloesser RJ McCammon J Chen G . Cellular mechanisms underlying the antidepressant effects of ketamine: Role of AMPA receptors. Biol Psychiatry. (2008) 63:349–52. doi: 10.1016/j.biopsych.2007.05.028, PMID: 17643398

[5] Koike H Iijima M Chaki S . Involvement of AMPA receptor in both the rapid and sustained antidepressant-like effects of ketamine in animal models. Behav Brain Res. (2011) 224:107–11. doi: 10.1016/j.bbr.2011.05.035, PMID: 21669235

[6] Li N Lee B Liu RJ Banasr M Dwyer JM Iwata M . mTOR-dependent synapse formation underlies the rapid antidepressant effects of NMDA antagonists. Science. (2010) 329:959–64. doi: 10.1126/science.1190287, PMID: 20724638 PMC3116441

[7] McCarthy B Bunn H Santalucia M Wilmouth C Muzyk A Smith CM . Dextromethorphan-bupropion (Auvelity) for the treatment of major depressive disorder. Clin Psychopharmacol Neurosci. (2023) 21:609–16. doi: 10.9758/cpn.23.1081, PMID: 37859435 PMC10591164

[8] Crewe HK Lennard MS Tucker GT Woods FR Haddock RE . The effect of selective serotonin re-uptake inhibitors on cytochrome P4502D6 (CYP2D6) activity in human liver microsomes. Br J Clin Pharmacol. (1992) 34:262–5. doi: 10.1111/j.1365-2125.1992.tb04134.x, PMID: 1389951 PMC1381398

[9] Preskorn SH Shah R Neff M Golbeck AL Choi J . The potential for clinically significant drug-drug interactions involving the CYP 2D6 system: effects with fluoxetine and paroxetine versus sertraline. J Psychiatr Pract. (2007) 13:5–12. doi: 10.1097/00131746-200701000-00002, PMID: 17242587

[10] Spina E Santoro V D’Arrigo C . Clinically relevant pharmacokinetic drug interactions with second-generation antidepressants: an update. Clin Ther. (2008) 30:1206–27. doi: 10.1016/S0149-2918(08)80047-1, PMID: 18691982

[11] Flockhart DA Thacker D McDonald C Desta Z . The flockhart cytochrome P450 drug-drug interaction table. Division Clin Pharmacology Indiana Univ School Med. (2021).

[12] Shin JG Soukhova N Flockhart DA . Effect of antipsychotic drugs on human liver cytochrome P-450 (CYP) isoforms *in vitro*: preferential inhibition of CYP2D6. Drug Metab Dispos. (1999) 27:1078–84. doi: 10.1016/S0090-9556(24)15029-5, PMID: 10460810

[13] Sandson NB Armstrong SC Cozza KL . An overview of psychotropic drug-drug interactions. Psychosomatics. (2005) 46:464–94. doi: 10.1176/appi.psy.46.5.464, PMID: 16145193

[14] Bousman CA Stevenson JM Ramsey LB Sangkuhl K Hicks JK Strawn JR . Clinical pharmacogenetics implementation consortium (CPIC) guideline for CYP2D6, CYP2C19, CYP2B6, SLC6A4, and HTR2A genotypes and serotonin reuptake inhibitor antidepressants. Clin Pharmacol Ther. (2023) 114:51–68. doi: 10.1002/cpt.2903, PMID: 37032427 PMC10564324

[15] Thase ME Youakim JM Skuban A . Efficacy and safety of dextromethorphan-bupropion in major depressive disorder: A randomized, double-blind, controlled trial. Am J Psychiatry. (2022) 179:490–9. 10.1176/appi.ajp.21080800 35582785

[16] Cohen SA Müller WE . Effects of piracetam on N-methyl-D-aspartate receptor properties in the aged mouse brain. Pharmacology. (1993) 47:217–22. doi: 10.1159/000139100, PMID: 8234409

[17] Gualtieri F Manetti D Romanelli MN Ghelardini C . Design and study of piracetam-like nootropics, controversial members of the problematic class of cognition-enhancing drugs. Curr Pharm Des. (2002) 8:125–38. doi: 10.2174/1381612023396582, PMID: 11812254

[18] Winblad B . Piracetam: A review of pharmacological properties and clinical uses. CNS Drug Rev. (2005) 11:169–82. doi: 10.1111/j.1527-3458.2005.tb00268.x, PMID: 16007238 PMC6741724

[19] Baek JH Jung S Son H Kang JS Kim HJ . Glutamine supplementation prevents chronic stress-induced mild cognitive impairment. Nutrients. (2020) 12:910. doi: 10.3390/nu12040910, PMID: 32224923 PMC7230523

[20] Son H Baek JH Go BS Jung DH Sontakke SB Chung HJ . Glutamine has antidepressive effects through increments of glutamate and glutamine levels and glutamatergic activity in the medial prefrontal cortex. Neuropharmacology. (2018) 143:143–52. doi: 10.1016/j.neuropharm.2018.09.040, PMID: 30266598

[21] Lin TY Yang TT Lu CW Wang SJ . Inhibition of glutamate release by bupropion in rat cerebral cortex nerve terminals. Prog Neuropsychopharmacol Biol Psychiatry. (2011) 35:598–606. doi: 10.1016/j.pnpbp.2010.12.029, PMID: 21216268

[22] Ye L Huang Y Zhao L Li Y Sun L Zhou Y . IL-1β and TNF-α induce neurotoxicity through glutamate production: a potential role for neuronal glutaminase. J Neurochem. (2013) 125:897–908. doi: 10.1111/jnc.12263, PMID: 23578284 PMC3747774

[23] Guerrero-Molina MP Morales-Conejo M Delmiro A Morán M Domínguez-González C Arranz-Canales E . High-dose oral glutamine supplementation reduces elevated glutamate levels in cerebrospinal fluid in patients with mitochondrial encephalomyopathy, lactic acidosis and stroke-like episodes syndrome. Eur J Neurol. (2023) 30:538–47. doi: 10.1111/ene.15626, PMID: 36334048

[24] Baek JH Park H Kang H Kim R Kang JS Kim HJ . The role of glutamine homeostasis in emotional and cognitive functions. Int J Mol Sci. (2024) 25:1302. doi: 10.3390/ijms25021302, PMID: 38279303 PMC10816396

[25] Cheung N . Clinical experience and optimisation of the Cheung glutamatergic regimen for refractory psychiatric diseases. (2025). doi: 10.20944/preprints202511.2246.v1, PMID: 41116858

[26] Cheung N . An oral ketamine-like approach to treatment-resistant obsessive-compulsive disorder—a review of mechanism, clinical experience, and future directions. (2025). doi: 10.20944/preprints202512.1958.v1, PMID: 41116858

